# Molecular characteristics and evaluation of the phenotypic detection of carbapenemases among *Enterobacterales* and *Pseudomonas* via whole genome sequencing

**DOI:** 10.3389/fcimb.2024.1357289

**Published:** 2024-07-04

**Authors:** Bingshao Liang, Yuou Chen, Zhuwei Liang, Xueying Li, Hao Cai, Hanyu Lai, Huamin Zhong, Yongqiang Xie, Lianfen Huang, Fei Gao, Yan Long

**Affiliations:** ^1^ Clinical Laboratory, Guangzhou Women and Children’s Medical Center, Guangzhou Medical University, Guangdong Provincial Clinical Research Center for Child Health, Guangzhou, China; ^2^ KingMed School of Laboratory Medicine, Guangzhou Medical University, Guangzhou, China; ^3^ Clinical Laboratory, Guangdong Provincial Second Hospital of Traditional Chinese Medicine (Guangdong Provincial Engineering Technology Research Institute of Traditional Chinese Medicine), Guangzhou, China; ^4^ Clinical Laboratory, The First People’s Hospital of Zhaoqing, Zhaoqing, China

**Keywords:** carbapenem-resistant gram-negative bacteria, carbapenemases, combined-disc tests, modified carbapenem inactivation method, whole genome sequencing

## Abstract

**Background/purpose(s):**

The continuously increasing carbapenem resistance within *Enterobacterales* and *Pseudomonas* poses a threat to public health, nevertheless, the molecular characteristics of which in southern China still remain limited. And carbapenemase identification is a key factor in effective early therapy of carbapenem-resistant bacteria infections. We aimed to determine the molecular characteristics of these pathogens and compare commercial combined disc tests (CDTs) with the modified carbapenem inactivation method (mCIM) and EDTA-CIM (eCIM) in detecting and distinguishing carbapenemases using whole genome sequencing (WGS).

**Methods:**

A total of 78 *Enterobacterales*, 30 *Pseudomonas* were obtained from two tertiary hospitals in southern China. Susceptibility tests were conducted using an automated VITEK2 compact system with confirmation via the Kirby–Bauer method. The WGS was conducted on all clinical isolates and the molecular characteristics were analyzed by screening the whole genome sequences. CDTs with or without cloxacillin, mCIM, and eCIM, were performed and compared by taking WGS results as the benchmark.

**Results:**

A total of 103 carbapenem non-susceptible and 5 carbapenem susceptible bacteria were determined, with *Klebsiella pneumoniae* (42.7%), *Pseudomonas aeruginosa* (23.3%) and *Escherichia coli* (18.4%) being most prevalent. Carbapenemase genes were detected in 58 (56.3%) of the 103 carbapenem-non-susceptible clinical isolates, including 46 NDM, 6 KPC, 3 IMP, 1 IPM+VIM,1NDM+KPC, and 1 OXA-181. Carbapenemase-producing isolates were detected more frequently in *Enterobacterales* (76.3%). Among *K. pneumoniae*, the major sequence types were st307 and st11, while among *E. coli* and *P. aeruginosa*, the most prevalent ones were st410 and st242 respectively. For carbapenemase detection in *Enterobacterales*, the mCIM method achieved 100.00% (95% CI, 92.13–100.00%) sensitivity and 94.44% (70.63–99.71%) specificity (kappa, 0.96); for *Pseudomonas*, detection sensitivity was 100% (5.46–100.00%), and 100% (84.50–100.00%) specificity (kappa, 0.65). Commercial CDT carbapenemase detection sensitivity for *Enterobacterales* was 96.49% (86.84–99.39%), and 95.24% (74.13–99.75%) specificity (kappa, 0.90); for *Pseudomonas*, carbapenemase detection sensitivity was 100.00% (5.46–100.00%) and 37.93% (21.30–57.64%) specificity (kappa, 0.04). When cloxacillin testing was added, CDT specificity reached 84.61% (64.27–94.95%).

**Conclusion:**

The molecular epidemiology of carbapenem-non-susceptible isolates from pediatric patients in Southern China exhibited distinctive characteristics. Both the mCIM–eCIM combination and CDT methods effectively detected and differentiated carbapenemases among *Enterobacterales* isolates, and the former performed better than CDT among *Pseudomonas*.

## 1 Introduction

Carbapenems are considered a last-line class of antibiotics used for the treatment of infections caused by multidrug-resistant gram-negative bacteria. Owing to the lack of effective and safe alternative treatment options, carbapenem-resistant (CR) gram-negative bacteria, including CR-*Enterobacterales* (CRE) and CR-*Pseudomonas aeruginosa* (CRPA) cause a wide range of infections in hospitals of all sizes, leading to significant morbidity and mortality (Reyes et al., 2023; Ding et al., 2024). Some of the novel antimicrobial agents have not been available for clinical use in China, adding further complexity to the issue (Tamma et al., 2022; Zeng et al., 2023). The World Health Organization has categorized these pathogens as “Critical” priority, emphasizing the urgent need for novel antibiotics (Paul et al., 2022). Moreover, genetic and phenotypic differences in CR gram-negative bacteria, which vary based on region and population, have not yet been elucidated (Jiang et al., 2023). It is reported that the most prevalent clone of carbapenem-resistant *K. pneumoniae* (CRKP) circulating in China is ST11, while for CR-*E. coli* (CREC) and CRPA, the prevalent clones were ST410 and ST463 respectively (Zhang et al., 2023; Ba et al., 2024; Hu et al., 2024). Carbapenemase production, a key resistance mechanism among these pathogens, causes severe and often deadly infections with a higher 30-day mortality (Wei et al., 2022; Reyes et al., 2023). These carbapenemases can be divided into three Ambler classes: class A (e.g., *Klebsiella pneumoniae* carbapenemase, KPC), class B (metallo β–lactamases, MBLs), and class D (OXA-48-like carbapenemase). Rapid detection and differentiation of these carbapenemases is critical for the initiation of effective therapy; the identification and classification of carbapenemases have significant therapeutic, epidemiological, and infection-control implications. The drug combination ceftazidime/avibactam can be given to patients infected with class A and some class D carbapenemase-producing bacteria, but not to those infected with bacteria producing class B carbapenemases (Zeng et al., 2023). Furthermore, although bacteria producing OXA-48-like carbapenemases can be tested as susceptible to carbapenems, they are often associated with carbapenem treatment failure (Boyd et al., 2022).

In clinical laboratories, the detection of carbapenemases, particularly of CRPA, is challenging. Several of the existing phenotypic methods for screening carbapenemases, such as the Modified Hodge test, have low sensitivity for New Delhi metallo-beta lactamase (NDM) producers and do not distinguish between carbapenemase types (Zhou et al., 2018). Since 2017, the Clinical & Laboratory Standards Institute (CLSI) has recommended the modified carbapenem inactivation method (mCIM) for detecting carbapenemases in CRE; the CLSI expanded the scope of mCIM to CRPA in 2018 (CLSI, 2017; CLSI, 2018). In the new version of the CLSI guideline launched in 2023, carbapenemase phenotype testing was further emphasized (CLSI, 2023). Since 2018, EDTA-CIM (eCIM) has been recommended for identifying MBLs in CRE (CLSI, 2018), although it is not recommended for distinguishing carbapenemase in CR *Pseudomonas* isolates. Furthermore, the mCIM and eCIM methods require a broth incubation process that can be cumbersome (CLSI, 2023).

Commercial combined-disc tests (CDTs), which were among the first tests used in clinical laboratories to detect carbapenemases in CRE, utilize chemical compounds to specifically inhibit carbapenemases from different Ambler classes. Phenylboronic acid (PBA) inhibits class A carbapenemases, and EDTA inhibits class B carbapenemases (Haider et al., 2022). A similar method has been reported for discriminating between KPC and MBLs in *Pseudomonas*, and the cloxacillin test was introduced to help exclude over-expressing isolates; nonetheless, these methods utilize different inhibitors and interpretive criteria (Lopez-Hernandez et al., 2020).

Most phenotypic methods for carbapenemase screening use polymerase chain reaction (PCR) results as the reference (Gill et al., 2020). However, as PCR traditionally targets specific genes, it may generate false negatives if specific carbapenemase genes are not targeted, especially when novel variant genes emerge (Voulgari et al., 2020). Whole genome sequencing (WGS) method provides more comprehensive results when it is caused either a novel carbapenemase or by an AmpC enzymes combined with reduced permeability due to the alteration or down-regulation of porins (Di Pilato et al., 2022).

To investigate the genomic population structure of these pathogens in southern region of China and to improve carbapenemase screening, we determined the molecular characteristics and evaluated the performance of the mCIM and eCIM combination methods, as well as the commercial CDT method, to detect and distinguish carbapenemases among gram-negative bacteria collected in two tertiary hospitals in southern China, using WGS.

## 2 Materials and methods

### 2.1 Bacterial strains

A total of 108 non-duplicate clinical isolates were collected from two medical centers in southern China from 2016 to 2023, primarily from pediatric patients. The isolates were identified via matrix-assisted laser desorption/ionization–time of flight (MALDI–TOF) mass spectrometry (MS) (Bruker Biotyper; Bruker Daltonik, Bremen, Germany). Susceptibility tests were conducted using an automated VITEK2 compact system (bioMérieux, Marcy l’Etoile, France), with confirmation via the Kirby–Bauer method. The breakpoint criteria were specified according to the latest CLSI guidelines (CLSI, 2023). ATCC27853 and ATCC25922 were used as the negative control. Carbapenem non-susceptible *Enterobacterales* were defined as those that were non-susceptible (with intermediate susceptibility or resistance) to imipenem, meropenem, or ertapenem. Carbapenem non-susceptible *Pseudomonas* were defined as those that were not susceptible to either imipenem or meropenem. The study was approved by the Ethics Committee of Guangzhou Women and Children’s Medical Center. Written informed consent was waived, for this study primarily concentrated on bacteria.

### 2.2 Genome-wide identification of carbapenemase genes

The total genomic DNA required for WGS was extracted using the SteadyPure Bacteria Genomic DNA Extraction Kit (Hunan Accurate Biotechnology Co., Ltd., Changsha, China). The WGS was conducted at the Beijing Genomics Institute (Beijing, China) via short-read sequencing. Data analysis was performed as described in the previous work (Liang et al., 2022). Carbapenemase genes were identified by screening the whole genome sequencing data against data from the Center for Genomic Epidemiology website (https://cge.food.dtu.dk), using ResFinder 4.1. ATCC27853 was used as the negative control.

### 2.3 Multi-locus sequence typing of carbapenem non-susceptible isolates

All the carbapenem non-susceptible *K. pneumoniae*, *E. coli* and *P. aeruginosa* isolates were subjected to multi-locus sequence typing through uploading the WGS data to Genomic Epidemiology website (https://cge.food.dtu.dk) and searched by MLST typing, or the STs were determined by searching against the MLST database (https://pubmlst.org). A phylogenetic tree was drawn for carbapenem-resistant *E. coli* isolates by BacWGSTdb website (http://bacdb.cn/BacWGSTdb) using WGS data.

### 2.4 CDT procedure

Fresh bacterial colonies were used to prepare a 0.5 McFarland turbidity suspension (0.45% saline). This suspension was uniformly streaked onto Mueller–Hinton (MH) agar plates. The CDT was performed according to the manufacturers’ recommendations (Zhuhai DL Biotech. Co., Ltd., Guangdong, China). Four test discs containing carbapenem were applied to each plate. Generally, imipenem was used, as per the CDT manufacturers’ recommendations. Other carbapenem was used to screen bacteria that are susceptible to imipenem. Then, PBA (5 μL, for class A carbapenemases), EDTA (5 μL, for class B carbapenemases), or both PBA and EDTA (5 μL each, for class A and B carbapenemases) were dispensed onto three of the four discs, with the control being one disc without any inhibitor, according to the manufacturer’s instructions. The plate was then incubated at 35°C for 18–24 h. The diameters of the growth inhibitory zones around the discs were then compared. If the inhibition zone around a disc containing the inhibitor had a diameter ≥5 mm larger than that around the control disc, the strain was considered to be positive for the respective carbapenemase classes (Zhang et al., 2022a). For the 30 *Pseudomonas* strains (including 3 negative controls), cloxacillin tests were performed to screen out the ampC β-lactamase-overproducing isolates (Pasteran et al., 2011). ATCC27853 was used as the negative control.

### 2.5 mCIM procedure

The mCIM tests for carbapenemase detection among gram-negative bacteria were performed following the CLSI guidelines. A loopful (1 μL) of *Enterobacterales* or a 10 μL loopful of *Pseudomonas* was plated in 2 mL of tryptic soy broth and vigorously mixed for 15 s. Thereafter, a carbapenem disc (as in the CDT) was added to the suspension using sterile forceps, followed by incubation at 35°C for 4 h. Shortly after incubation, *Escherichia coli* ATCC25922 suspension (0.5 McFarland turbidity) was spread on MH agar. The immersed disc was then removed from the bacterial suspension; the excess liquid was expelled from the disc, and it was placed onto the inoculated MH agar and incubated overnight at 35°C. The inhibition zone diameter was then measured. The results were interpreted according to the CLSI criteria (CLSI, 2023). ATCC27853 was used as the negative control.

### 2.6 eCIM procedure

We used the eCIM and mCIM tests in combination to differentiate class B carbapenemase from serine carbapenemases in gram-negative bacteria. However, this approach is valid only when the mCIM is positive. The eCIM test was performed following the CLSI guidelines and prior literature. We added EDTA solution (20 μL, 0.5 M) to prepare a 2 mL tryptic soy broth solution, with a final concentration of 5 mM EDTA. The other procedures were the same as they were for the mCIM method. An increase in zone diameter ≥5 mm relative to that of mCIM was considered positive for a class B carbapenemase producer; otherwise, serine carbapenemases were recorded (Kumari et al., 2021; CLSI, 2023).

### 2.7 Statistical analysis

Test sensitivity and specificity were analyzed (with 95% confidence intervals, CIs) using the free software VassarStats (http://vassarstats.net). The concordance of the results of the two tests to those of the reference standard method was assessed by calculating the Kappa coefficient (κ) using SPSS 27.0 (SPSS, Inc., Chicago, IL, USA).

## 3 Results

### 3.1 Clinical characterization of 108 isolates included in two large tertiary hospitals in southern region of China

Overall, 103 carbapenem non-susceptible gram-negative bacteria and 5 carbapenem-susceptible gram-negative bacteria were included. The isolates were collected from sputum (n = 32), midstream urine (n = 33), blood (n = 11), catheter (n = 5), stool (n = 5), and other sites (n = 17). Of the 103 carbapenem non-susceptible isolates, 76 belonged to *Enterobacterales*, 27 belonged to *Pseudomonas. K. pneumoniae* was counting for 42.7%, while *P. aeruginosa* made up 23.3% and *E. coli* 18.4%. Of these 103 isolates, 90.3% were resistant to carbapenem.

### 3.2 Prevalence and distribution of carbapenemase genes among 103 carbapenem non-susceptible isolates

Carbapenemase genes were detected in 58 (56.3%) of the 103 carbapenem non-susceptible gram-negative bacterial isolates, and it were more frequently detected in the *Enterobacterales* (76.3%). The most frequently detected carbapenemase gene in *Enterobacterales* was *bla*NDM (61.8%), which was even more frequent in *K. pneumoniae* (at 63.6%) and *E. coli* (at 84.2%). Carbapenemase genes were substantially less prevalent among the *Pseudomonas* strains, occurring in only 3.70% of the *Pseudomonas* isolates tested. The carbapenemase genes identified included subtypes *bla*NDM (*bla*NDM-1, *bla*NDM-5), *bla*KPC-2, *bla*OXA-181, *bla*IMP (*bla*IMP-4, *bla*IMP-8, *bla*IMP-26, *bla*IMP-38), and *bla*VIM (*bla*VIM-2, *bla*VIM-46) (**Table 1**). Co-occurrence of *bla*NDM-1 with *bla*KPC-2, and of *bla*IMP-8 with *bla*VIM-2 and *bla*VIM-46, were observed.

**Table 1:** Characterization of 103 carbapenem non-susceptible gram-negative bacteria.

Microorganism		No. (%)	NDM	KPC	IMP	OXA-48-like	NDM+KPC	IMP+VIM	No detected carbapenemase
*Enterobacterales* (n=76)	*K.pneumoniae*	44 (42.7)	27 (26.2%)	6 (5.8%)	3 (2.9%)	1 (1.0%)	1 (1.0%)		6 (5.8%)
	*E. coli*	19 (18.4)	16 (15.5%)						3 (2.9%)
	*E. cloacae*	5 (4.9)							5 (4.9%)
	*Salmonella*	3 (2.9)	1 (1.0%)						2 (1.9%)
	*Proteus*	2 (1.9)							2 (1.9%)
	*Enterobacteria*	2 (1.9)	1 (1.0%)						1 (1.0%)
	*C. freundii*	1 (1.0)	1 (1.0%)						
*Pseudomonas* (n=27)	*P. aeruginosa*	24 (23.3)							24 (23.3%)
	*P. putida*	1 (1.0)						1 (1.0%)	
	other	2(1.9)							2 (1.9%)

NDM, New Delhi metallo-β-lactamase; KPC, Klebsiella pneumoniae carbapenemase; IMP, imipenemase; VIM, verona integron-mediated metallo-β-lactamase; OXA-48-like, oxacillinase-48-like carbapenemase.

### 3.3 The molecular characteristics of 103 carbapenem non-susceptible isolates

**Figure 1:**
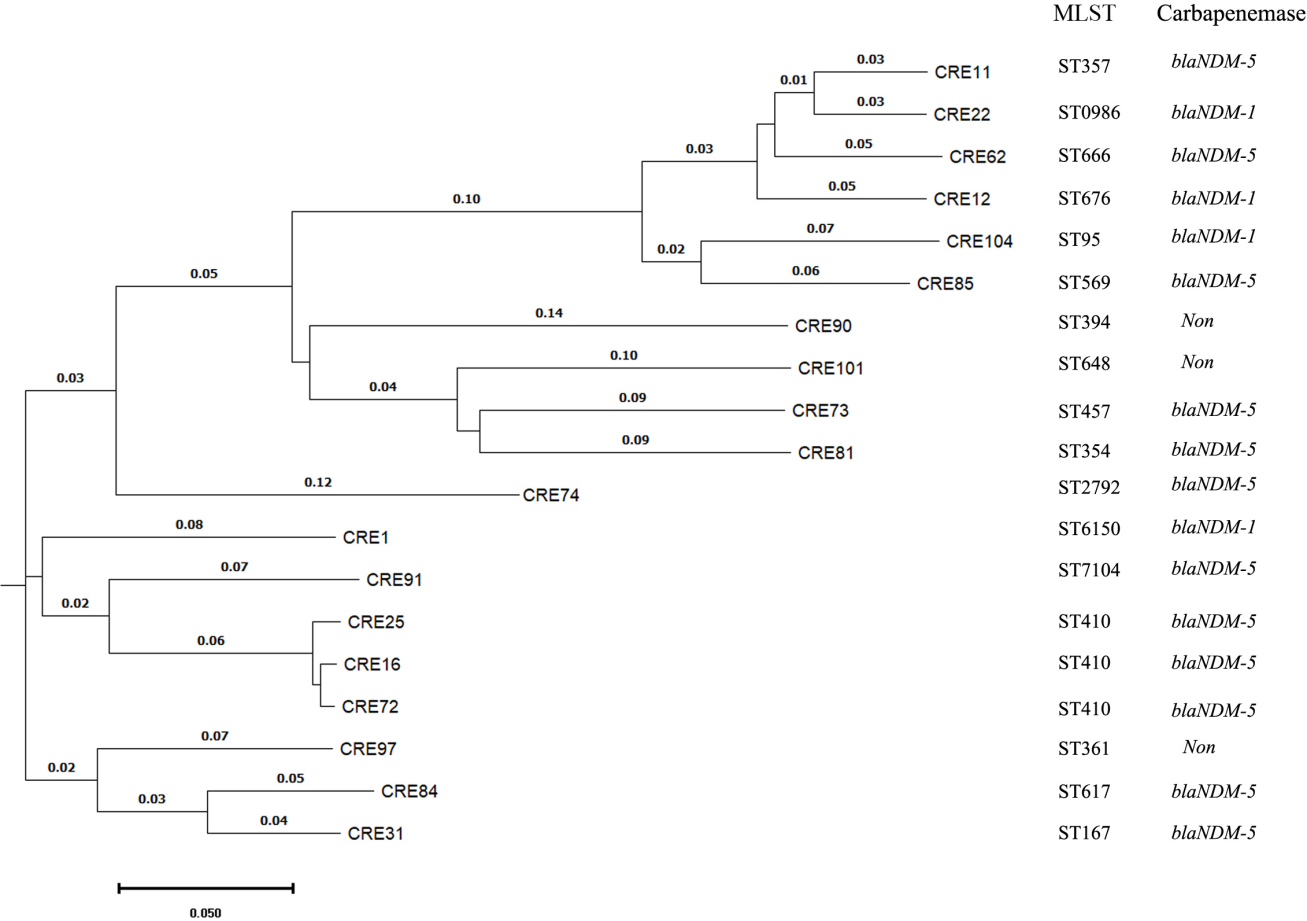
The phylogenetic tree constructed for carbapenem-resistant *E. coli* isolates through the utilization of whole genome sequencing by BacWGSTdb. MLST means multi-locus sequence typing.

All the carbapenem non-susceptible *K. pneumoniae*, *E. coli* and *P. aeruginosa* isolates were subjected to multi-locus sequence typing. A total of 25 STs were identified in *K. pneumoniae*, with st307 and st11 being the most prevalent, collectively accounting for 27.3% of the isolates. Among *E. coli*, 17 STs were detected, and st410 was the most common, representing 15.8%. Similarly, in *P. aeruginosa*, 16 STs were identified, and the top three STs were st242, st244 and st385, accounting for 37.5% of the isolates. The phylogenetic tree constructed for carbapenem-resistant *E. coli* isolates, as depicted in **Figure 1**, revealed that the isolates were grouped into three distinct clades, with the hypervirulent CREC st410 clone occupying a prominent position within clade II.

### 3.4 CDT performance

The commercial CDT successfully detected and distinguished carbapenemases among the *Enterobacterales* isolates evaluated (**Tables 2**, **3**), achieving 96.49% sensitivity (95% CI, 86.84–99.39%) and 95.24% specificity (95% CI, 74.13–99.75%) (kappa, 0.90) for both the detection and classification tests. However, for carbapenemase detection among the *Pseudomonas* isolates, the CDT achieved 100.00% sensitivity (95% CI,5.46–100.00%) but only 37.93 specificity (95% CI,21.30–57.64%) (kappa, only 0.04). When cloxacillin testing was included to discriminate (screen out) ampC β-lactamase-overproducing isolates, specificity reached 84.61% (95% CI, 64.27–94.95%) (kappa, 0.26).

**Table 2:** The phenotypic methods for detecting and distinguishing carbapenemases among 108 Gram-Negative Bacteria.

Microorganism	Nos.	mCIM			eCIM		EDTA		APB		EDTA+APB	
		Pos	Ind	Neg	Pos	Neg	Pos	Neg	Pos	Neg	Pos	Neg
***Enterobacterales***	78											
Carbapenemases	57	57	0	0	49	8	48	9	6	51	55	2
NDM	46	46	0	0	46	0	46	0	0	46	46	0
KPC	6	6	0	0	0	6	0	6	6	0	6	0
IMP	3	3	0	0	3	0	2	1	0	3	2	1
OXA-48-like	1	1	0	0	0	1	0	1	0	1	0	1
NDM+KPC	1	1	0	0	0	1	0	1	0	1	1	0
No carbapenemase	19	1	3	15	1	18	0	19	1	18	1	18
Carbapenem-susceptible	2	0	0	2	0	2	0	2	0	2	0	2
***Pseudomonas***	30											
Carbapenemases	1	1	0	0	1	0	1	0	0	1	1	0
IMP+VIM	1	1	0	0	1	0	1	0	0	1	1	0
No carbapenemase	26	0	1	24^*^	0	25^*^	1	25	17	9	18	8
Carbapenem-susceptible	3	0	0	3	0	3	0	3	2	1	2	1

Ind: indeterminate; *: one isolate was missed when tested with mCIM and eCIM.

**Table 3:** Overall sensitivity and specificity of phenotype diagnostic assays.

Diagnostic Assays		Sensitivity		Specificity		Kappa
		%	95% CI	%	95% CI	
Enterobacterales						
detection tests	mCIM	100.00	92.13-100.00	94.44	70.63-99.71	0.96
	CDT	96.49	86.84-99.39	95.24	74.13-99.75	0.90
classification tests	mCIM+eCIM	98.24	89.37-99.91	94.44	70.63-99.71	0.93
	CDT	96.49	86.84-99.39	95.24	74.13-99.75	0.90
Pseudomonas						
detection tests	mCIM+eCIM	100.00	5.46-100.00	100.00	84.50-100.00	0.65
	CDT	100.00	5.46-100.00	37.93	21.30-57.64	0.04
	CDT+CLOX	100.00	5.46-100.00	84.61	64.27-94.95	0.26

### 3.5 mCIM–eCIM combination performance

The mCIM–eCIM combination testing successfully detected and distinguished carbapenemases in the *Enterobacterales* isolates (**Tables 2**, **3**). For detection, mCIM–eCIM achieved 100.00% sensitivity (95% CI, 92.13–100.00%) and 94.44% specificity (95% CI, 70.63–99.71%) (kappa, 0.96). For classification, mCIM–eCIM achieved 98.24% sensitivity (95% CI, 89.37–99.91%), with the same specificity as in the detection test. Even among the *Pseudomonas* evaluated, the mCIM–eCIM carbapenemase detection test achieved 100% sensitivity (95% CI,5.46–100.00%) and 100% specificity (95% CI, 84.50–100.00%) (kappa, 0.65). (**Table 2**).

## 4 Discussion

Monitoring the molecular epidemiology of carbapenem-non-susceptible isolates is key for controlling the spread these pathogens and detecting and distinguishing carbapenemases is crucial for clinicians when selecting appropriate antibiotic treatment (Tamma et al., 2022). To investigate the genomic population structure of carbapenem-non-susceptible isolates and to improve carbapenemase screening, we determined the molecular characteristics of these pathogens and evaluated the performance of the combined mCIM–eCIM method and commercial CDT method against the WGS.

We detected carbapenemases in 76.3% of the *Enterobacterales* isolates; this frequency is approximately the same as that of another epidemiology study of carbapenem non-susceptible *Enterobacterales* conducted from 2017 to 2019 in German (von Laer et al., 2022). However, the *bla*NDM carbapenemase gene, detected here among the *K. pneumoniae* isolates, is reported to occur much more frequently in strains from pediatric patients than in those from adult patients (Lee et al., 2022). Most of the CR-*Pseudomonas* isolates identified here were not carbapenemase-producing, unlike those identified in pediatric patients from other parts of China, South America, and Central America (Patil et al., 2023; Reyes et al., 2023). The carbapenem-resistance pattern observed here was similar to that observed in the USA (Reyes et al., 2023).

The genomic population structure of carbapenem-non-susceptible isolates varied based on region and population. In this study, the most prevalent carbapenem non-susceptible *K. pneumoniae* clones were st307 and st11. and the latter of which were all *bla*KPC-2 producing strains from adult patients at the First People’s Hospital of Zhaoqing. As depicted in literatures, the st11-*bla*KPC-2 clone is the predominant clones circulating among adult patients in China (Hu et al., 2020). Meanwhile, our findings reveal that the st307 clone was exclusively detected among pediatric patients, it may emerge as a major CRKP clones among pediatric patients in southern China, as reported in another children’s center in Shenzhen (Patil et al., 2021). However, although their STs was identical, they differed in their carbapenemase patterns, specifically, featuring metallo-β-lactamases in this study. Among *P. aeruginosa* in this study, the top three STs were st242, st244 and st385, which differed from most carbapenemase producing isolates among adult patients in other regions of China (Li et al., 2023). So, in this study, the molecular epidemiology of carbapenem non-susceptible isolates exhibited distinctive characteristics that are significant for understanding their prevalence and transmission.

The mCIM-eCIM combination test reportedly performs well against carbapenemase-producing *Pseudomonas* isolates, but not against imipenemase- and Sao Paulo metallo-β-lactamase-producing strains (Gill et al., 2020). Thus, in this study, we evaluated the performance of mCIM–eCIM combination test mainly using 29 non-carbapenemase-producing CR *Pseudomonas* clinical strains from southern China. The mCIM–eCIM combination exhibited excellent and reliable carbapenemase detection among the *Enterobacterales* and *Pseudomonas* isolates, with high sensitivity and specificity. Nonetheless, we obtained indeterminate results for some of the non-carbapenemase-producing strains, including CRE and CRPA isolates. The eCIM test’s classification ability may be hampered when class A and class B carbapenemases are coproduced because strains that achieve this coproduction effectively hydrolyze the substrates regardless of whether EDTA is added (Hao et al., 2022).

In China, the commercial CDT test has been widely used for *Enterobacterales*, while its use for *Pseudomonas* isolates has only recently been reported in a few studies (Zhang et al., 2022b). Here, the CDT accurately detected and distinguished carbapenemases in *Enterobacterales*. By using PBA and EDTA separately and simultaneously, the CDT test was also able to detect and classify carbapenemase coproduction in *Enterobacterales*. However, the CDT produced a false positive when used to test CRPA with *Pseudomonas* derived cephalosporinase, a type of chromosomal cephalosporinase ampC β-lactamase hyperproduction. The robustness of the CDT was substantially improved by including a cloxacillin test to exclude the influence of ampC β-lactamase hyperproduction when the CDT result was positive. The lower value of 95% CI of sensitivity of the CDT could have been higher had more carbapenemase-producing strains been included in this study, however the performance of the CDT with cloxacillin testing was no better than that of the mCIM–eCIM combination test among *Pseudomonas* isolates. We observed a strain of *Enterobacter cloacae* with *bla*CMH, a type of ampC β-lactamase produced false positive result with CDT test either. When we use CDT to detect the carbapenemase, if the strain often produces Class C β–lactamases, the cloxacillin test could be routinely carried out even among *Enterobacterales* isolates.

WGS can detect all kinds of carbapenemase genes, known or unknown, as with the co-occurrence of *bla*VIM-2, *bla*VIM-46 and *bla*IMP-8 in the *Pseudomonas putida* strain that we observed. In addition, another study found that some *bla*KPC-2 variants could not be detected using PCR (Ding et al., 2021). Co-occurring strains like this could contribute to the hidden dissemination of bacteria. Consequently, WGS, which more comprehensively identifies carbapenemase genes among CR gram-negative bacteria, may provide a better gold standard.

## 5 Conclusion

The molecular epidemiology of carbapenem-non-susceptible isolates from pediatric patients in Southern China displayed distinct characteristics. Both the mCIM–eCIM combination and CDT effectively detected and differentiated carbapenemases among the *Enterobacterales* isolates, whereas the former performed better than CDT among the *Pseudomonas* isolates. This novel attempt may thus improve the rapid and accurate detection and identification of carbapenemases, improving both therapy and infection control.

## Data availability statement

The original contributions presented in the study are included in the article/supplementary material. Further inquiries can be directed to the corresponding author.

## Author contributions

BL: Funding acquisition, Writing – original draft. YC: Methodology, Writing – original draft. ZL: Methodology, Writing – original draft. XL: Methodology, Writing – original draft. HC: Methodology, Writing – original draft. HL: Methodology, Writing – original draft. HZ: Data curation, Writing – original draft. YX: Formal Analysis, Writing – original draft. LH: Funding acquisition, Writing – original draft. FG: Writing – original draft, Software. YL: Conceptualization, Writing – review & editing.
